# Comorbidity Between Depression and Anxiety in Adolescents: Bridge Symptoms and Relevance of Risk and Protective Factors

**DOI:** 10.1007/s10862-021-09880-5

**Published:** 2021-03-30

**Authors:** Deniz Konac, Katherine S. Young, Jennifer Lau, Edward D. Barker

**Affiliations:** 1 Department of Psychology, Institute of Psychiatry, Psychology & Neuroscience, King’s College London, 16 De Crespigny Park, Camberwell, London, SE5 8AB UK; 2 Department of Psychology, Adana Alparslan Turkes Science and Technology University, Adana, Turkey; 3 Social, Genetic & Developmental Psychiatry Centre, Institute of Psychiatry, Psychology and Neuroscience, King’s College London, London, UK

**Keywords:** Depression, Anxiety, Comorbidity, Network analysis, Adolescent, Avon longitudinal study of parents and children (ALSPAC)

## Abstract

Depression and anxiety are highly prevalent and comorbid in adolescents, and this co-occurrence leads to worse prognosis and additional difficulties. The relationship between depression and anxiety must be delineated to, in turn, reduce and prevent the comorbidity, however our knowledge is still limited. We used network analysis to investigate bridge symptoms; symptoms that connect individual depression and anxiety symptoms and thus can help explain the comorbidity. We also examined the role of relevant risk and protective factors in explaining these symptom-level associations between these disorders. We analyzed data from the Avon Longitudinal Study of Children and Parents (*n* = 3670). Depression and anxiety symptoms, peer victimization, bullying, peer relational problems, prosocial behavior, and parental monitoring were assessed at a single time point around age 13 years. Stressful life events (SLEs) were assessed at age 11 years. We identified the most prominent bridge symptoms among depression (“feeling unhappy”, “feeling lonely”) and anxiety symptoms (“worrying about past”, “worrying about future”). Peer relational difficulties and SLEs were strongly associated with several depression and anxiety symptoms, such that these two risk factors created a link between individual depression and anxiety symptoms. Prosocial behavior had several negative associations with symptoms of both disorders, suggesting it can be an important protective factor.

**Supplementary Information:**

The online version contains supplementary material available at 10.1007/s10862-021-09880-5.

## Introduction

Depression and anxiety are highly common in adolescents, with a 12-month prevalence of 10% of any mood disorders and 24.9% of any anxiety disorders in adolescents in the US (Kessler et al. 2012), and are often comorbid (Garber and Weersing 2010). This comorbidity causes further debilitation than depression or anxiety alone; it results in higher suicidality, poorer prognosis, worse treatment outcomes, lower life satisfaction, more physical health problems, less likelihood to attend college, greater overall impairment, and academic difficulties (Newman et al. 1998; Cummings et al. 2014; Schoevers et al. 2005). As such, prevention and reduction of comorbidity is important, yet at the same time, necessitates a deep understanding of the inter-relationship between depression and anxiety (Jones et al. 2019). Importantly, the mechanisms that underlie this co-occurrence, to date, are poorly understood (Karlsson et al. 2006). Delineating symptom to symptom relationships and the relevance of risk and protective factors might constitute a sound step toward understanding these mechanisms.

The network approach is considered a particularly useful method to investigate comorbidity; it conceptualizes comorbidity as resulting from mutual interactions among symptoms (Cramer et al. 2010). Symptoms that are highly associated with both depression and anxiety can be thought of as bridge symptoms, as they are considered active and reinforcing between the two disorders (Fried et al. 2017). Bridge symptoms are thus thought to contribute to the development and maintenance of comorbidity between disorders (Cramer et al. 2010). As a result, network modelling allows for identifying specific pathways, at the level of symptoms, through which disorders interact with each other (Cramer et al. 2010). This implies that interventions targeting bridge symptoms may help reduce comorbidity (Borsboom and Cramer 2013).

A handful of network studies examined the associations specifically between depression and generalized anxiety symptoms. These studies showed that several bridge edges (i.e. associations between two symptoms belonging to different disorders) existed between the disorders. The results from two adult samples (Cramer et al. 2010; Beard et al. 2016) and one adolescent sample (McElroy et al., 2018) were comparable in that the depression symptom of “sad mood” and anxiety symptom of “excessive worrying” were among the most influential bridge symptoms. Depression symptom of “guilt” was also a bridge symptom in two of these studies (McElroy et al., 2018; Beard et al. 2016). However, a common limitation across these studies was that some of the items in the measures were highly similar either within the same measure (e.g. anxiety symptoms of “too much worry” and “unable to control worry”, or the depression symptoms of “trouble sleeping” and “sleeps less than most children”) or between measures (e.g. symptom of “feeling restless” being measured in both anxiety and depression measures). An overlap in symptoms can artificially inflate edge weights and centrality indices (Beard et al. 2016).

Assessing how relevant risk or protective factors can affect symptom networks and bridge symptoms is also important (Fried and Cramer 2017). Cramer et al. (2010) suggested that “etiological factors” such as stressful life events can provide insight into the complex mechanisms underlying bridge symptoms between anxiety and depression. However, only a few studies included risk factors in network models to date (Pereira-Morales et al. 2019; Schellekens et al. 2020). For example, Schellekens et al. (2020) investigated how risk and protective factors were interconnected with symptoms of depression, a fatigue sum score and one anxiety symptom (“I felt fearful”) in cancer patients. Their results showed that risk factors of helplessness and physical symptoms were positively associated with depression symptoms and fatigue. They also found that the protective factor of illness acceptance was negatively associated with the anxiety symptom and depression symptoms. These results shed light on the pathway to depression in cancer patients. Similarly, Pereira-Morales et al. (2019) examined the associations between different types of childhood maltreatment, personality traits, and a few selected depression and anxiety symptoms. Their results indicated that sexual abuse in childhood was associated with anxiety symptoms of being unable to relax and feelings of tingle. Emotional neglect in childhood was positively associated with anxiety symptoms of worrying and feelings of tingle, and negatively associated with the personality trait of openness. While these studies examined some depression and anxiety symptoms together with other psychological distress items, no study to date investigated how relevant risk factors and protective factors interacted with depression and anxiety symptoms specifically, or the role these factors played in relation with comorbidity of these disorders.

In the present research, we included risk factors that affect both anxiety and depression symptoms. We examined these risks factors at 13 years of age, as early adolescence is a common age of onset for anxiety and depression symptoms (Kessler et al. 2007) and adolescent-onset is associated with poorer prognosis and stronger impairment (Copeland et al. 2014; Dunn and Goodyer 2006). We examined peer victimization, bullying peers, low quality of peer relationships, and experiencing stressful life events (Stapinski et al. 2015; Ivarsson et al. 2005; Oppenheimer and Hankin 2011; Young and Dietrich 2015). We also included protective factors for the onset of anxiety and depression. These factors included parental knowledge of child whereabouts, adolescent disclosure/discourse with caregivers about their whereabout, and prosocial behaviors towards peers and adults (Garthe et al. 2015; Hamza and Willoughby 2011; La Greca and Harrison 2005).

Therefore, we aimed to explore, by network analytic methods, which bridge symptoms and risk/protective factors play an important role in the co-occurrence of depression and anxiety in a large community sample of adolescents. We then explored how the initial network changed in terms of structure and bridge centrality statistics after introducing several risk/protective factors. We thus aimed to examine how risk/protective factors contribute to high levels of comorbidity between depression and anxiety in adolescents (Garber and Weersing 2010). Based on previous research (McElroy et al., 2018; Beard et al. 2016; Cramer et al. 2010), we expected depression symptoms related to “guilt” and “sad mood” to be among the most influential bridge symptoms. Since GAD worry symptoms have not previously been examined in this context, we did not have a priori hypotheses regarding the GAD worry symptoms with the highest bridge properties. In addition, we expected all of the risk factors included (e.g. peer victimization/bullying, peer relational problems, and SLEs) would have several associations with symptoms of both disorders since these are well-established risk factors for depression and anxiety in youth (Stapinski et al. 2015; Oppenheimer and Hankin 2011; Young and Dietrich 2015). Finally, in line with prior studies (La Greca and Harrison 2005; Garthe et al. 2015) we hypothesized the protective factors (e.g. prosocial behavior and parental monitoring) would have negative associations with the symptoms of depression and anxiety.

## Method

### Participants

Data was drawn from an ongoing epidemiological cohort study, the Avon Longitudinal Study of Children and Parents (ALSPAC). The cohort consists of 14,541 pregnant women residing in southwest England, who gave birth between 1st April 1991 and 31st December 1992 (13,988 children alive at 1 year). The sample size was increased to 15,454 pregnancies (15,589 fetuses) when the oldest child was 7 years of age by including additional eligible participants. 14,901 of these participants were alive at 1 year. The participants, who are still being followed, were broadly representative of the United Kingdom’s general population at the time of data collection (Boyd et al. 2013; Fraser et al. 2013).

A subsample (*n* = 3670) of this ALSPAC sample was used for the current study. The subsample consists of children who had available data on depression, anxiety, relational and overt victimization, relational and overt bullying behavior, peer relational problems, SLEs, prosocial behavior, and parental monitoring around 13 years of age.

Ethical approval for data collection for ALSPAC was obtained from the ALSPAC Ethics and Law Committee and the Local Research Ethics Committees (see http://www.bris.ac.uk/alspac/researchers/research-ethics), and informed consent for the use of data collected via questionnaires and clinics was obtained from participants following the recommendations of the ALSPAC Ethics and Law Committee at the time. Please note that the study website contains details of all the data that is available through a fully searchable data dictionary and variable search tool (http://www.bristol.ac.uk/alspac/researchers/our-data/).

### Measures

#### Depression Symptoms

Child depression symptoms at 13 years and 1 month of age were assessed using the parent reported version of the Short Mood and Feelings Questionnaire (SMFQ) (Angold et al. 1995). The SMFQ has 13 items that measure the extent to which the child exhibited depression symptoms (“feeling unhappy/miserable”, “not enjoying anything at all”, “feeling so tired and sitting around doing nothing”, “feeling very restless”, “feeling self is no good anymore”, “crying a lot”, “finding it hard to think properly/concentrate”, “hating self”, “feeling like a bad person”, “feeling lonely”, “thinking nobody really loves them”, “thinking self could never be as good as other kids”, “feeling self does everything wrong”) in the past two weeks (recoded as 1 = not true, 2 = sometimes true, 3 = true). It has been previously demonstrated that the SMFQ has high validity in capturing depression in the ALSPAC sample (Turner et al. 2014).

#### GAD Worry Symptoms

Child GAD worry symptoms at 13 years and 10 months were assessed using generalized anxiety disorder related items in the parent completed version of Development and Wellbeing Assessment (DAWBA). DAWBA is consisted of a set of questionnaires, interviews, and rating techniques that generate DSM-IV and ICD-10 psychiatric diagnoses on children and has been shown to have high validity for the population samples from the UK (Goodman et al. 2000; Ford et al. 2003). ALSPAC administered nine generalized anxiety disorder related items from DAWBA to parents when the study child was 13 years old. Due to the significant overlap of some of these anxiety items with depression items (e.g. “feeling restless”, “difficulty concentrating”, “feeling tired”, etc.), only the items regarding the content of the worry were included in the analyses. These items measure the amount the child worried about a variety of things (past behavior, schoolwork, disasters, own health, bad things happening to others, the future) in the past 6 months (1 = no, not at all, 2 = sometimes, 3 = often). Participants who replied to a previous skip question and reported that the study child never worries were recoded as though they replied “no, not at all” to the following generalized anxiety disorder items.

#### Peer Victimization and Bullying

Child bullying and victimization experiences at 12 years and 6 months were assessed using a shortened version of the Bullying and Friendship Interview Schedule (Wolke et al. 2001). The interviews were conducted by trained psychologists with the child and measured the frequency of overt and relational victimization behavior directed to child by peers as well as the frequency of overt and relational bullying behavior directed to peers by child over the past 6 months (1 = seldom, 2 = frequently/ >4 times, 3 = very frequently/ > once a week). Participants who reported not experiencing the situation being asked were recoded as having a score of 0 for the relevant question. Overt victimization category is consisted of five items (“had personal belongings taken”, “been threatened/blackmailed”, etc.), relational victimization category is consisted of four items (“others wouldn’t play with them to upset them”, “been made to do things didn’t want to”, etc.), overt bullying category is consisted of five items (“threatened/blackmailed anyone”, “hit/beaten anyone up”, etc.), and relational bullying category is consisted of four items (“told lies/nasty stories about them”, “spoilt games just to upset them”, etc.).

#### Peer Relational Problems

Peer relational problems at 13 years and 1 month of age were assessed using peer problems subscale of the parent reported version of the Strengths and Difficulties Questionnaire (SDQ)(Goodman 1997), which has been shown to have satisfactory levels of validity and reliability (Goodman 2001). SDQ is a brief behavioral screening questionnaire designed to use with 3 to 16-year-olds and it asks about 25 items that constitute five subscales. The peer problems subscale is consisted of five items (“rather solitary, tends to play alone”, “has at least one good friend”, “generally liked by other children”, “picked on or bullied by other children”, “gets on better with adults than with other children”) that ask about the child’s peer relationships in the last 6 months. Item “picked on or bullied by other children” was excluded from the analyses to minimize item overlap with the Bullying and Friendship Interview. “Has at least one good friend” and “generally liked by other children” items were reverse coded in order that higher scores for all items represent more severe peer problems (recoded as 0 = not true, 1 = somewhat true, 2 = certainly true).

#### Stressful Life Events (SLEs)

Life events since the child’s 9th birthday were assessed at 134 months (11.16 years) using a 47-item life event questionnaire completed by the mother (Jensen et al. 2015). Thirty three out of 47 items are utilized as they reflected negative life events (“respondent’s husband/partner died since study child’s 9th birthday”, “one of respondent’s children has died since study child’s 9th birthday”, etc.). Scores are recoded in order that participants who did not experience the event being asked are given a score of 0, participants who experienced the event either at between 9 to 10 years of age or since age 11 years are given a score of 1, and participants who experienced the event both at between 9 to 10 years of age and since age 11 years are given a score of 2.

#### Prosocial Behavior

Prosocial behavior at 13 years and 1 month of age was assessed by the parent reported version of the SDQ (Goodman 1997). The prosocial subscale is consisted of five items (“helpful if someone is hurt, upset or feeling ill”; “kind to younger children”, etc.) that ask about the child’s engagement in prosocial behavior in the last 6 months (recoded as 0 = not true, 1 = somewhat true, 2 = certainly true).

#### Parental Monitoring

Parental monitoring at 12 years and 6 months was assessed using the child reported parental monitoring questionnaire developed by ALSPAC based on Stattin and Kerr’s (2000) Parental Monitoring Questionnaire. Previous research has shown that this questionnaire loads onto four separate subscales for an ALSPAC subsample at age 14 and subscales of “parental knowledge” and “child disclosure” were found to have strong correlations with SMFQ scores (Pesola et al. 2015). Parental knowledge subscale is consisted of 10 items (“frequency carers know what teenager does in free time”, “frequency carers know what teenager spends money on”, etc.) and child disclosure subscale is consisted of 5 items (“frequency teenager keeps secrets from carers about what they do in free time”,” frequency teenager keeps things from carers about what they do nights/weekends”). Items measuring unawareness of parents were reverse coded in order that higher scores for all items represent higher levels of parental monitoring (recoded as 0 = never, 1 = hardly ever, 2 = sometimes, 3 = most of the time, 4 = always).

### Included Vs. Not-Included Sample

Participants who did not provide an answer to more than two items of any measure used in the study were removed from the current analysis sample. 112 participants did not provide enough data on depression measures, 192 on anxiety measures, 429 on overt victimization measures, 452 on relational victimization measures, 437 on overt bullying measures, 453 on relational bullying measures, 122 on peer relational problems measures, 122 on prosocial behavior measures, 1518 on child disclosure measures, and 1666 on parental knowledge measures. SLEs were measured at 11 years of age and 152 out of 7596 participants who had provided any data at all at the given time point did not provide enough data for this measure. The remaining participants who provided enough data on all of these measures were included in the study, resulting in a final sample size of 3670 (51.6% of total ALSPAC sample at age 13, *n* = 7108). Detailed explanation on the pattern of missingness can be found in the Supplementary Materials and is also visually presented in Supplementary Figure 1 (S1).We tested whether there were differences between those excluded and included on mother’s highest level of education, mother’s socioeconomic status, facing financial difficulties during pregnancy, and whether the mother lived with a partner during pregnancy. We found that participants with lower levels of maternal socioeconomic status (OR = 0.9, CI = 0.86, 0.94, *p* = 0.00) and whose parents faced financial difficulties during pregnancy (OR = 0.74, CI = 0.63, 0.88, *p* = 0.001) were less likely to be included in the analyses while participants with higher maternal education levels (OR = 1.29, CI = 1.23, 1.35, p = 0.00) were more likely to be included in the analyses.

### Statistical Analysis

A two-step approach was adopted. In Step-1, the network structure of depression and GAD worry symptoms alone were examined. Depression symptoms and GAD worry symptoms were specified as two different communities when performing bridge centrality calculations. Communities are groups of predefined nodes and are used to estimate bridge centrality indices (Jones et al. 2019). In Step-2, measures for peer victimization, bullying, peer problems, prosocial behavior, parental monitoring, and SLEs were included in the network. Three different communities were specified for depression symptoms (Depression), GAD worry symptoms (Anxiety), and SLEs scores (Stressful Events). Parental knowledge and child disclosure scores were combined into a fourth community (Parental Monitoring). Overt victimization score, relational victimization score, overt bullying score, relational bullying score, peer problems score, and prosocial behavior score were combined into a fifth community (Peer Relationships).

#### Network Estimation

All statistical analyses were performed using R (R Core Team 2019) version 3.6.0. An unregularized Gaussian graphical model (GGM; Costantini et al. 2015) was used to estimate the networks at both steps, as regularization is not needed with high sample size and low-dimensional settings (p < n), and unregularized models may be more efficacious in returning the true network model (Williams and Rast 2019). The networks were computed using the ggmModSelect function from R package qgraph (Epskamp et al. 2012) version 1.6.4, which chooses the best GGM according to Bayesian information criterion. Spearman’s correlation matrix was calculated for variables in the networks and partial correlations were estimated based on these correlations. In such partial correlation networks, the associations (i.e. edges) between two variables (i.e. nodes) indicate conditional dependence relations. Accordingly, if an edge exists between two nodes, this means these nodes are associated after controlling for all other nodes in the network (Schellekens et al. 2020). Finally, the algorithm used to plot the networks aims to place highly correlated nodes closer together, and nodes with fewer or weaker correlations are placed in peripherals of the graph (Fruchterman and Reingold 1991).

#### Node Centrality

Four centrality indices were estimated to examine the overall importance of each node in the networks (Opsahl et al. 2010). Four bridge centrality indices were estimated to examine how connected each node was with nodes in communities other than its own (Jones et al. 2019). R packages qgraph version 1.6.4 and networktools (Jones 2017) version 1.2.2. were used for the estimations.

Recently developed index of bridge expected influence (1-step) (BEI) was of special interest given it distinguishes between positive and negative edges unlike other commonly used indices. BEI sums up the edge weights from a node to all other nodes from communities other than the node’s own community. Because BEI does not take the absolute value of the edge weights, it can gauge the node’s cumulative influence on overall network activation (Robinaugh et al. 2016), and was shown to have the highest robustness at high sample sizes among other bridge centrality indices (Jones et al. 2019). The top 20% scoring nodes on BEI centrality index are colored and labelled as “bridge” nodes in both networks (Jones et al. 2019). The edge widths of networks in both steps are visually comparable given the same maximum value of edge width (0.65) was used when plotting the networks.

#### Stability of Networks and Node Centrality

R package bootnet (Epskamp et al. 2018b) version 1.3 was employed to assess the stability of network parameters. Accuracy of the edge weights was estimated by running 2500 nonparametric bootstraps and constructing 95% confidence intervals (CI) around each edge in the network (Epskamp et al. 2018b). Significance tests provided by this package were also employed to test whether any given edge was stronger than other edges in the network.

Correlation stability coefficients (CS-coefficient) were also calculated with the bootnet package to assess the stability of centrality and bridge centrality indices. CS-coefficients were calculated by running 2500 case-dropping subset bootstraps and represent what proportion of participants can be dropped from the analysis such that the correlation between the original centrality indices and the new ones are not lower than 0.7. Only centrality and bridge centrality indices found to have a CS-coefficient higher than the suggested cut off score of 0.25 were reported and interpreted (Epskamp et al. 2018b). Significance tests provided by this package were also employed to test differences between centrality indices for each node.

## Results

### Descriptive Statistics

The sample is consisted of 3670 participants (50.7% female). Frequencies of depression and anxiety symptoms are presented in Table 1 and descriptive statistics of sum scores are presented in Table 2.

**Table 1:** Frequencies of depression and anxiety symptoms

Symptom	Score of 1	Score of 2	Score of 3
Depression Symptoms			
“unhappy”: has felt unhappy/miserable	2029	1278	122
“enjoy”: hasn’t enjoyed anything at all	2982	406	38
“tired”: has felt so tired they sat around and did nothing	2531	813	87
“restless”: has felt very restless	2528	852	49
“no good”: has felt they were no good anymore	3123	270	37
“crying”: has cried a lot	3167	244	20
“concent.”: has found it hard to think properly/concentrate	2772	604	55
“hating”: has hated themselves	3230	188	12
“bad”: has felt they were a bad person	3275	144	9
“lonely”: has felt lonely	2900	504	24
“love”: has thought nobody really loved them	3124	274	31
“worse”: has thought they could never be as good as other kids	2992	393	44
“wrong”: felt they did everything wrong	2825	564	39
Anxiety Symptoms			
“past”: worries a lot about past behavior	2723	676	29
“school”: worries a lot about school	1849	1413	168
“dssters”: worries a lot about disasters	2897	504	27
“health”: worries a lot about their own health	2902	485	39
“others”: worries a lot about bad things happening to others	2595	781	48
“future”: worries a lot about the future	2591	780	49

**Table 2:** Descriptive statistics of risk variable sum scores

Risk Factor	Mean	Standard Deviation	Median	Minimum	Maximum
“ovt_vic”: overt victimization sum score	1.31	1.91	1	0	14
“rlt_vic”: relational victimization sum score	0.54	1.19	0	0	10
“ovt_bly”: overt bullying sum score	0.56	1.17	0	0	13
“rlt_bly”: relational bullying sum score	0.15	0.58	0	0	7
“peer_pr”: SDQ peer problems subscale sum score	1.07	1.49	1	0	10
“pro_soc”: SDQ prosocial subscale sum score	8.27	1.68	9	1	10
“disclsr”: child disclosure subscale sum score	14.09	3.64	14	0	20
“knowldg”: parental knowledge subscale sum score	26.05	4.29	27	3	32
“SLE”: SLEs sum score	2.88	2.59	2	0	21

### Step-1

**Fig. 1:**
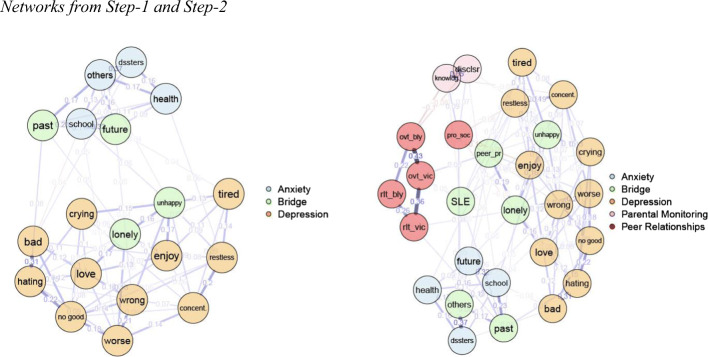
**a**) Depression and anxiety symptoms network in Step-1. **b**) Depression and anxiety symptoms, peer victimization, bullying, peer problems, prosocial behaviour, parental monitoring, and SLEs network in Step-2

**Fig. 2:**
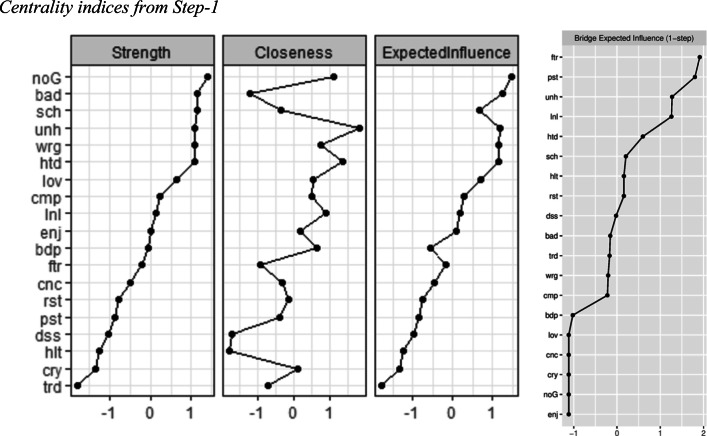
Centrality and bridge centrality indices for depression and anxiety symptoms network in Step-1

The network is presented in Panel-A in Fig. 1. Based on the 95% bootstrapped CI, the edge weights appeared rather stable (S2). Indices of strength, closeness, expected influence, and BEI were found to be stable and are presented in Fig. 2. Depression and GAD worry symptoms were more densely connected within their own communities, and their centrality values (i.e. indices depicting relative importance of the symptoms in the network) were comparable. GAD worry symptoms of “worrying about bad things happening to others” and “worrying about school”, and depression symptoms of “feeling self is no good anymore” and “feeling unhappy” had the highest node strength values in the network, meaning these had the highest number and magnitude of direct associations with other symptoms. Further details regarding the most central symptoms can be found in Supplementary Materials.

#### Bridge Symptoms

The top 20% scoring nodes on BEI are colored in green and labelled as “bridge” in the network in Panel-A in Fig. 1.

Out of these highest-ranking bridge nodes, the most prominent bridge symptoms among GAD worry items were “worrying about past” and “worrying about future.” As can be seen in Panel-A in Fig. 1, GAD worry symptom of “worrying about past” had edges with depression symptoms of “hating self” and “feeling lonely.” GAD worry symptom of “worrying about future” had edges with depression symptoms of “feeling lonely”, “feeling tired”, and “feeling like doing everything wrong.”

The most prominent bridge symptoms among depression items were “feeling unhappy” and “feeling lonely.” Depression symptom of “feeling unhappy” had edges with GAD worry symptoms of “worrying about school” and “worrying about bad things happening to others.” Depression symptom of “feeling lonely” had edges with GAD worry symptoms of “worrying about past” and “worrying about future” as reported above. The list of all such bridge edges between depression and GAD worry symptoms are listed in Table 3.

**Table 3:** List and comparison of bridge edges in Step-1 and Step-2

Bridge Edges	Step-1	Step-2
Hating self -- Worrying about past	Y	Y
Feeling unhappy -- Worrying about school	Y	Y
Feeling very restless -- Worrying about health	Y	Y
Feeling lonely -- Worrying about past	Y	Y
Feeling self is a bad person -- Worrying about disasters	Y	Y
Feeling lonely -- Worrying about future	Y	Y
Thinking self cannot be as good as others -- Worrying about school	Y	Y
Feeling self is a bad person -- Worrying about school	Y	Y
Feeling unhappy -- Worrying about bad things happening to others	Y	Feeling unhappy -- SLEs -- Worrying about bad things happening to others
Feeling so tired they sat around and did nothing -- Worrying about future	Y	Feeling so tired they sat around and did nothing -- Peer relational problems -- Worrying about future
Feeling self did everything wrong -- Worrying about future	Y	N
Feeling like self did everything wrong -- Worrying about health	N	Y

Finally, relevant CS-coefficients indicating the stability of the centrality and bridge centrality indices (S3), significant differences between symptom rankings on these indices (S4-S7), and significant differences between edge weights (S8) can be found in Supplementary Materials.

### Step-2

**Fig. 3:**
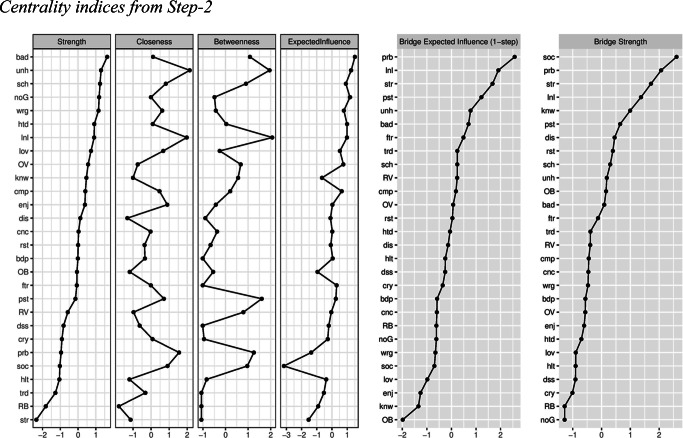
Centrality and bridge centrality indices for depression and anxiety symptoms, peer victimization, bullying, peer problems, prosocial behaviour, parental monitoring, and SLEs network in Step-2

The network is presented in Panel-B in Fig. 1. Based on the 95% bootstrapped CI, the edge weights were very stable (S9). Indices of strength, closeness, betweenness, expected influence, bridge strength, and BEI were also stable and are presented in Fig. 3. Depression symptoms, GAD worry symptoms, victimization and bullying measures, and parental monitoring measures were more densely connected within their own communities. Depression symptoms of “feeling self is no good anymore” and “feeling unhappy”, and GAD worry symptoms of “worrying about bad things happening to others” and “worrying about school” had the highest node strength values in the network, replicating the findings in Step-1. Overt victimization was the highest-ranking risk factor and parental knowledge was the highest-ranking protective factor on node strength index. Further details regarding the most central symptoms and risk/protective factors can be found in Supplementary Materials.

#### Bridge Symptoms

The top 20% scoring nodes on BEI are colored in green and labelled as “bridge” in the network in Panel-B in Fig. 1.

With the addition of the risk and protective factors in this step, results of Step-1 both remained the same and changed. With regard to remaining the same, the edges between depression symptom of “feeling unhappy” and GAD worry symptom of “worrying about school”, and between depression symptom of “feeling lonely” and GAD worry symptoms of “worrying about past” and “worrying about future” remained largely unchanged. The edges between GAD worry symptom of “worrying about past” and depression symptoms of “hating self” and “feeling lonely” still existed in this step. In addition, “feeling unhappy” and “feeling lonely” remained the most influential bridge symptoms from the depression community, and “worrying about past” remained the most influential bridge symptom from the GAD worries community. “Worrying about bad things happening to others” became a bridge symptom as influential as “worrying about future.” Even though BEI values of “worrying about bad things happening to others” and “worrying about future” were comparable, a closer examination of the edges these nodes had with nodes outside of GAD worries community suggests some differences. “Worrying about bad things happening to others” had stronger bridge edges with the SLEs and prosocial behavior, rather than with depression symptoms. This indicates that “worrying about bad things happening to others” was influential in associating risk/protective factors and GAD worry symptoms with each other, but not in directly associating GAD worry symptoms and depression symptoms. On the other hand, “worrying about future” ranked as high as “worrying about bad things happening to others” on BEI and still had bridge edges with depression symptoms including “feeling lonely.” These results suggest “worrying about future” was still more influential in associating GAD worries and depression than “worrying about bad things happening to others.” A new bridge edge between the depression symptom of “feeling like self did everything wrong” and GAD worry symptom of “worrying about health” emerged. The list of these bridge edges and how they changed compared to the bridge edges in Step-1 can be found in Table 3.

#### Other Bridge Nodes

Peer relational problems and SLEs were the most prominent risk factors that acted as bridge nodes in linking certain depression and GAD worry symptoms. For example, GAD worry symptom “worrying about future” and depression symptom “feeling tired” became associated via peer relational problems. Similarly, GAD worry symptom of “worrying about bad things happening to others” and depression symptom of “feeling unhappy” became associated via SLEs. Following SLEs and peer relational problems, overt and relational victimization were the risk factors that emerged as moderately influential bridge nodes.

SLEs had edges with depression symptoms of “feeling unhappy”, “feeling restless”, “feeling tired”; and GAD worry symptoms of “worrying about past” and “worrying about bad things happening to others.” Peer relational problems had edges with depression symptoms of “feeling lonely”, “thinking self cannot be as good as others”, “feeling tired” and “finding it hard to think properly/concentrate”, and GAD worry symptoms of “worrying about past” and “worrying about future.”

Prosocial behavior emerged as the most prominent protective factor. This node ranked the highest in bridge strength, indicating it had the highest number of associations with the rest of the nodes in the network. However, it ranked extremely low on BEI, indicating most of these associations were negative, as also can be seen in the network. Prosocial behavior was negatively associated with depression symptoms of “feeling lonely”, “feeling like doing everything wrong”, “not enjoying anything at all”, and “feeling restless.” Interestingly, the only positive association between prosocial behavior and the symptoms was with GAD worry symptom of “worrying about bad things happening to others.” Prosocial behavior also had positive associations with other protective factors from parental monitoring community.

Finally, relevant CS-coefficients indicating the stability of the centrality and bridge centrality indices (S10), significant differences between symptom rankings on these indices (S11-S16), and significant differences between edge weights (S17) can be found in supplementary materials. The most central risk/protective factors were discussed in Supplementary Materials.

## Discussion

In this study, we aimed to examine bridge symptoms between depression and non-overlapping anxiety symptoms (i.e. GAD worry symptoms) as these bridges can say a lot about how one disorder is actively associated with – or can innervate –another disorder. In addition, we explored the risk (and protective) factors that may have facilitated (and impeded) these associations. While this study is the first to specifically examine the bridge symptoms of depression and anxiety in adolescents, we based our expectations on existing network research on adults and adolescents (Beard et al. 2016; Cramer et al. 2010, McElroy et al., 2018). This study is also one of the first in this line of research to show that certain risk factors played an important role in the comorbidity of depression and anxiety as well as to identify an influential protective factor. These results contribute to the existing knowledge of comorbidity between depression and anxiety in three main ways.

Firstly, in Step-1, we identified the most influential bridge symptoms for depression (“feeling unhappy”, “feeling lonely”) and GAD worries (“worrying about past”, “worrying about future”). It is important to note that even though “feeling lonely” is not a depression symptom listed in the versions of the DSM, it has been included in several other depression questionnaires as a theoretically relevant item (e.g. Child Behavior Check List, Birleson’s Depression Self Rating Scale, and Children’s Depression Scale). Previously, research has found other non-DSM symptoms to be more influential than some of the DSM symptoms (Fried et al. 2016) and assessing the importance of non-DSM depression symptoms were advised (Fried and Nesse 2015). Our results support the idea that examining relevant non-DSM depression symptoms might reveal more insights into the nature of depression. Overall, the said most influential bridge symptoms remained essentially unchanged in Step-2, suggesting these symptoms may be maintaining comorbidity between depression and anxiety by innervating inter-disorder associations (Cramer et al. 2010), even after controlling for the effects of risk factors and protective factors included in Step-2.

The cross-sectional nature of this study precludes interpretating the directionality of the bridge pathways (Boschloo et al. 2016). Accordingly, the edges present in this study could be reflecting unidirectional relationships of either direction or bidirectional relationships which were previously shown to exist between depression and anxiety (Jacobson and Newman 2017). Hence, future research will want to explore directional inferences in network models. To this end, the edges between depression symptoms of “hating self” and “feeling lonely” and GAD worry symptom of “worrying about past” may be interpreted as worrying about past events leading one to have negative feelings toward themselves, as well as to feel lonely, which might represent an important pathway to comorbidity. Equally, feeling lonely may result in negative feelings about the self and prompt worries about past behavior that have led to this. Interestingly, rumination, a process involving perseveratively thinking about one’s problems and their causes, is also found to strongly associate with hating self (Flett et al. 2020) and can even be involved in a loss of social support (Nolen-Hoeksema et al. 2008). In addition, as rumination increases, the association between depressed and anxious mood gets stronger (Starr and Davila 2011). Accordingly, this finding is in line with the existing literature given the close relationship between rumination and worries about the past.

Secondly, we examined the role of several risk factors and protective factors with regard to these bridge symptoms. Peer relational problems and SLEs were the risk factors that exhibited the highest bridge properties. For example, GAD worry symptom of “worrying about future” and depression symptom of “feeling tired” were directly associated in Step-1, but they became associated via peer relational problems in Step-2. Accordingly, it is possible that peer relational problems can act as a common cause for depression and GAD worry symptoms; or that the associations between these symptoms were facilitated by this risk factor (Epskamp and Fried 2018).

In addition to facilitating relationships between symptoms, a risk factor can influence the comorbidity between the disorders due to having several associations with multiple symptoms of both mental health problems. Here, peer relational problems was strongly associated to depression symptoms related to feelings of loneliness and low self-esteem; and the GAD worry symptom of “worrying about future.” This may be interpreted as having problems with peers leading to both feelings of loneliness/worthlessness and worries about the future, hence adding to the direct association between these symptoms and contributing to comorbidity. Conversely, having peer problems due to having more severe depression/anxiety symptoms is equally plausible. Indeed, previous research indicated depression and anxiety symptoms may lead to peer relational problems (Oppenheimer and Hankin 2011) and vice versa (La Greca and Harrison 2005) without being able to provide insight into which specific symptoms might play the most important role in this phenomenon.

SLEs showed similar results as peer relational problems. Anxiety symptom of “worrying about bad things happening to others” and depression symptom of “feeling unhappy” became associated via SLEs in Step-2. This may be interpreted as experiencing SLEs may lead one to both experience low mood and worry about the safety of the loved ones. That is plausible given SLEs include loved ones getting harmed and one might worry about experiencing a similar event in the future and feel unhappy. SLEs also had edges with multiple depression symptoms of “feeling unhappy”, “feeling restless”, “feeling tired”; and GAD worry symptoms of “worrying about past” and “worrying about bad things happening to others.” Thus, SLEs may lead to exacerbations in all of these symptoms, some of which also inter-related with each other. Accordingly, in line with previous research (Young and Dietrich 2015; Van Veen et al. 2013), it may be hypothesized that experiencing SLEs exacerbates symptoms of both disorders and contributes to comorbidity by functioning like a common cause.

Thirdly, our results showed that prosocial behavior acted as a protective factor; it was negatively associated with a large number of depression and GAD worry symptoms. This indicates engaging in prosocial behavior may function as an important protective factor by decreasing symptom severity (Kramer et al. 2014), or that individuals less frequently engage in prosocial behavior as symptom severity increases (Broeren et al. 2013). Prosocial behavior was also positively associated with other protective factors of parental knowledge and child disclosure, which may be enhancing its protective effect indirectly. Interestingly, prosocial behavior was positively associated with GAD worry symptom of “worrying about bad things happening to others.” That suggests while engaging in prosocial behavior and caring about others is generally a protective factor, this characteristic might also predispose one to worry about safety of others.

Our results have several clinical implications. Depression symptoms of “feeling lonely” and “feeling unhappy”, and GAD worry symptoms of “worrying about past” and “worrying about future” emerged as the most important symptoms in enabling and maintaining the comorbidity between depression and anxiety. Accordingly, targeting these bridge symptoms, thus preventing the interactions between the disorders, may be a good strategy to treat comorbid depression and anxiety (Fried et al. 2017). Similarly, our results suggest that peer relational problems and SLEs were highly influential in contributing to the co-occurrence of depression and anxiety. Hence, experiencing these may put adolescents in higher risk of developing comorbid depression and anxiety. Thus, prevention of peer relational problems and providing early intervention to adolescents who experienced peer relational problems and SLEs may lead to decreases in comorbidity.

Finally, our findings should be considered in light of a number of limitations. Firstly, network analysis has been criticized on a number of grounds, including failure to replicate within and between samples, being non-causal, and that edges can result by chance (Forbes et al. 2017a; b; Steinley et al. 2017; Forbes et al. 2021). However, these critics themselves, have been criticized, on a number of grounds, including estimating unsuitable network models for the given data, using different network models that result in different network structures, and ignoring the impact of sampling variability on the results of network models (Borsboom et al. 2017; Epskamp et al. 2018a, b; Fried et al. 2020; Jones et al. 2020). Secondly, the data analyzed here is cross-sectional as these risk and protective factors, and symptoms of anxiety and depression were present together only at age 13. Additional research should test if the associations identified here replicate in longitudinal network analysis that spans early- to late-adolescence. Thirdly, the variability of the items was low due to the low symptom and risk factor endorsements of the non-clinical sample, which was argued to possibly influence edge strengths (Terluin et al. 2016). Finally, most of the measures used were parent-reported and that might have limited the capacity to capture the true states of the psychological constructs.

These limitations notwithstanding, our study is first to specifically examine bridge symptoms/edges between depression and anxiety in a large community sample of adolescents and to investigate how risk and protective factors are relevant to these associations. Examining these associations around 13 years of age may be particularly informative given age of onset of depression and anxiety is in early adolescence (Kessler et al. 2007) and the associations around this time point may be setting in place the patterns for long term comorbidity. Thus, our findings provide first steps for understanding symptom to symptom associations between depression and anxiety, while also considering prominent risk/protective factors.

## Supplementary Information

ESM 1 (DOCX 2103 kb)

## Data Availability

Data is available via the ALSPAC study, if access is granted by the ALSPAC committee.

## References

[CR1] Angold A, Costello EJ, Messer SC (1995). Development of a short questionnaire for use in epidemiological studies of depression in children and adolescents. International Journal of Methods in Psychiatric Research.

[CR2] Beard C, Millner AJ, Forgeard MJC, Fried EI, Hsu KJ, Treadway MT, Björgvinsson T (2016). Network analysis of depression and anxiety symptom relationships in a psychiatric sample. Psychological Medicine.

[CR3] Borsboom D, Cramer AOJ (2013). Network analysis: An integrative approach to the structure of psychopathology. Annual Review of Clinical Psychology.

[CR4] Borsboom D, Fried EI, Epskamp S, Waldorp LJ, van Borkulo CD, van der Maas HLJ, Cramer AOJ (2017). False alarm? A comprehensive reanalysis of “evidence that psychopathology symptom networks have limited replicability” by Forbes, Wright, Markon, and Krueger (2017). Journal of Abnormal Psychology.

[CR5] Boschloo, L., Schoevers, R. A., van Borkulo, C. D., Borsboom, D., & Oldehinkel, A. J. (2016). The network structure of psychopathology in a community sample of preadolescents. *Journal of Abnormal Psychology, 125*(4), 599–606. 10.1037/abn0000150 27030994

[CR6] Boyd A, Golding J, Macleod J, Lawlor DA, Fraser A, Henderson J, Molloy L, Ness A, Ring S, Davey Smith G (2013). Cohort profile: The ‘children of the 90s’—The index offspring of the Avon longitudinal study of parents and children. International Journal of Epidemiology.

[CR7] Broeren S, Muris P, Diamantopoulou S, Baker JR (2013). The course of childhood anxiety symptoms: Developmental trajectories and child-related factors in Normal children. Journal of Abnormal Child Psychology.

[CR8] Copeland WE, Angold A, Shanahan L, Costello EJ (2014). Longitudinal patterns of anxiety from childhood to adulthood: The Great Smoky Mountains study. Journal of the American Academy of Child and Adolescent Psychiatry.

[CR9] Costantini G, Epskamp S, Borsboom D, Perugini M, Mõttus R, Waldorp LJ, Cramer AOJ (2015). State of the aRt personality research: A tutorial on network analysis of personality data in R. Journal of Research in Personality.

[CR10] Cramer AOJ, Waldorp LJ, van der Maas HLJ, Borsboom D (2010). Comorbidity: A network perspective. The Behavioral and Brain Sciences.

[CR11] Cummings CM, Caporino NE, Kendall PC (2014). Comorbidity of anxiety and depression in children and adolescents: 20 years after. Psychological Bulletin.

[CR12] Dunn V, Goodyer I (2006). Longitudinal investigation into childhood-and adolescence-onset depression: Psychiatric outcome in early adulthood. British Journal of Psychiatry.

[CR13] Epskamp S, Fried EI (2018). A tutorial on regularized partial correlation networks. Psychological Methods.

[CR14] Epskamp S, Cramer AOJ, Waldorp LJ, Schmittmann VD, Borsboom D (2012). Qgraph: Network visualizations of relationships in psychometric data. Journal of Statistical Software.

[CR15] Epskamp, S., Fried, E. I., Borkulo, C. D., Van, Robinaugh, D. J., Marsman, M., Dalege, J., Rhemtulla, M., & … Ramer, A. O. J. (2018). Investigating the utility of fixed-margin sampling in network psychometrics. *Multivariate Behavioral Research*, 1–15. 10.1080/00273171.2018.1489771. 10.1080/00273171.2018.1489771 30463456

[CR16] Epskamp S, Borsboom D, Fried EI (2018). Estimating psychological networks and their accuracy: A tutorial paper. Behav Res.

[CR17] Flett, G.L., Burdo, R. & Nepon, T. (2020). Mattering, Insecure Attachment, Rumination, and Self-Criticism in Distress Among University Students. *Int J Ment Health Addiction*. 10.1007/s11469-020-00225-z.

[CR18] Forbes MK, Wright AGC, Markon KE, Krueger RF (2017). Evidence that psychopathology symptom networks have limited replicability. Journal of Abnormal Psychology.

[CR19] Forbes MK, Wright AGC, Markon KE, Krueger RF (2017). Further evidence that psychopathology networks have limited replicability and utility: Response to Borsboom et al. (2017) and Steinley et al. (2017). Journal of Abnormal Psychology.

[CR20] Forbes, M. K., Wright, A. G. C., Markon, K. E., & Krueger, R. F. (2021). On Unreplicable Inferences in Psychopathology Symptom Networks and the Importance of Unreliable Parameter Estimates. *Multivariate Behavioral Research*, 1–9. 10.1080/00273171.2021.1886897. 10.1080/00273171.2021.1886897 PMC8654099 33599559

[CR21] Ford T, Goodman R, Meltzer H (2003). The British child and adolescent mental health survey 1999: The prevalence of DSM-IV disorders. Journal of the American Academy of Child and Adolescent Psychiatry.

[CR22] Fraser A, Macdonald-Wallis C, Tilling K, Boyd A, Golding J, Davey Smith G, Henderson J, Macleod J, Molloy L, Ness A, Ring S, Nelson SM, Lawlor DA (2013). Cohort profile: The Avon longitudinal study of parents and children: ALSPAC mothers cohort. International Journal of Epidemiology.

[CR23] Fried, E. I., & Cramer, A. O. J. (2017). Moving forward: challenges and directions for psychopathological network theory and methodology. *Perspect Psychol Sci, 12*(6), 999–1020. 10.1177/1745691617705892. 10.1177/1745691617705892 28873325

[CR24] Fried EI, Nesse RM (2015). Depression sum-scores don’t add up: Why analyzing specific depression symptoms is essential. BMC Medicine.

[CR25] Fried EI, Epskamp S, Nesse RM, Tuerlinckx F, Borsboom D (2016). What are “good” depression symptoms? Comparing the centrality of DSM and non-DSM symptoms of depression in a network analysis. Journal of Affective Disorders.

[CR26] Fried EI, van Borkulo CD, Cramer AOJ, Boschloo L, Schoevers RA, Borsboom D (2017). Mental disorders as networks of problems: A review of recent insights. Social Psychiatry and Psychiatric Epidemiology.

[CR27] Fried, E. I., van Borkulo, C. D., & Epskamp, S. (2020). On the importance of estimating parameter uncertainty in network psychometrics: a response to Forbes et al. (2019). *Multivariate Behav Res*, 1–6. 10.1080/00273171.2020.1746903. 10.1080/00273171.2020.1746903 32264714

[CR28] Fruchterman TMJ, Reingold EM (1991). Graph drawing by force-directed placement. Software Pract. Exp..

[CR29] Garber J, Weersing VR (2010). Comorbidity of anxiety and depression in youth: Implications for treatment and prevention. Clinical Psychology: Science and Practice.

[CR30] Garthe, R. C., Sullivan, T., & Kliewer, W. (2015). Longitudinal relations between adolescent and parental behaviors, parental knowledge, and internalizing behaviors among urban adolescents. *J Youth Adolesc, 44*(4):819–832. 10.1007/s10964-014-0112-0. 10.1007/s10964-014-0112-0 PMC5826787 24609843

[CR31] Goodman R (1997). The strengths and difficulties questionnaire: A research note. Journal of Child Psychology and Psychiatry and Allied Disciplines.

[CR32] Goodman R (2001). Psychometric properties of the strengths and difficulties questionnaire. Journal of the American Academy of Child and Adolescent Psychiatry.

[CR33] Goodman R, Ford T, Richards H, Gatward R, Meltzer H (2000). The development and well-being assessment: Description and initial validation of an integrated assessment of child and adolescent psychopathology. Journal of Child Psychology and Psychiatry.

[CR34] Hamza CA, Willoughby T (2011). Perceived parental monitoring, adolescent disclosure, and adolescent depressive symptoms: A longitudinal examination. Journal of Youth and Adolescence.

[CR35] Ivarsson T, Broberg AG, Arvidsson T, Gillberg C (2005). Bullying in adolescence: Psychiatric problems in victims and bullies as measured by the youth self report (YSR) and the depression self-rating scale (DSRS). Nordic Journal of Psychiatry.

[CR36] Jacobson NC, Newman MG (2017). Anxiety and depression as bidirectional risk factors for one another: A meta-analysis of longitudinal studies. Psychological Bulletin.

[CR37] Jensen, S. K., Dickie, E. W., Schwartz, D. H., Evans, C. J., Dumontheil, I., Paus, T., & Barker, E. D. (2015). Effect of early adversity and childhood internalizing symptoms on brain structure in young men. *JAMA Pediatr, 169*(10):938–946.10.1001/jamapediatrics.2015.1486. 10.1001/jamapediatrics.2015.1486 PMC5137198 26280410

[CR38] Jones, P, J., (2017). Networktools: Tools for Identifying Important Nodes in Networks. R Package Version 1.1.0. 〈https://CRAN.R-project.org/package=networktools〉.

[CR39] Jones, P. J., Ma, R., & McNally, R. J. (2019). Bridge centrality: a network approach to understanding comorbidity. *Multivariate Behav Res*, 1–15. 10.1080/00273171.2019.1614898. 10.1080/00273171.2019.1614898 31179765

[CR40] Jones, P. J., Williams, D. R., & McNally, R. J. (2020). Sampling variability is not nonreplication: A Bayesian reanalysis of Forbes, Wright, Markon, and Krueger. *Multivariate Behavioral Research*, 1–7. 10.1080/00273171.2020.1797460 32731766

[CR41] Karlsson L, Pelkonen M, Ruuttu T, Kiviruusu O, Heilä H, Holi M, Marttunen JM (2006). Current comorbidity among consecutive adolescent psychiatric outpatients with DSM-IV mood disorders. European Child & Adolescent Psychiatry..

[CR42] Kessler RC, Amminger GP, Aguilar-Gaxiola S, Alonso J, Lee S, Ustün TB (2007). Age of onset of mental disorders: A review of recent literature. Current Opinion in Psychiatry.

[CR43] Kessler RC, Avenevoli S, Costello EJ, Georgiades K, Green JG, Gruber MJ, He JP, Koretz D, McLaughlin K, Petukhova M, Sampson NA, Zaslavsky AM, Merikangas KR (2012). Prevalence, persistence, and Sociodemographic correlates of DSM-IV disorders in the National Comorbidity Survey Replication Adolescent Supplement. Archives of General Psychiatry.

[CR44] Kramer TJ, Caldarella P, Young KR, Fischer L, Warren JS (2014). Implementing strong kids school-wide to reduce internalizing behaviors and increase Prosocial behaviors. Education and Treatment of Children.

[CR45] La Greca AM, Harrison HM (2005). Adolescent peer relations, friendships, and romantic relationships: Do they predict social anxiety and depression?. Journal of Clinical Child and Adolescent Psychology.

[CR46] McElroy, E., Fearon, P., Belsky, J., Fonagy, P., Patalay, P. (2018). Networks of depression and anxiety symptoms across development. *Journal of the American Academy of Child & Adolescent Psychiatry, 57*(2), 964–973. 10.1016/j.jaac.2018.05.027. 10.1016/j.jaac.2018.05.027 PMC6290121 30522742

[CR47] Newman DL, Moffitt TE, Caspi A, Silva PA (1998). Comorbid mental disorders: Implications for treatment and sample selection. Journal of Abnormal Psychology.

[CR48] Nolen-Hoeksema, S., Wisco, B. E., & Lyubomirsky, S. (2008). Rethinking rumination. *Perspect Psychol Sci, 3*(5):400–424. 10.1111/j.1745-6924.2008.00088.x. 10.1111/j.1745-6924.2008.00088.x 26158958

[CR49] Oppenheimer CW, Hankin BL (2011). Relationship quality and depressive symptoms among adolescents: A short-term multiwave investigation of longitudinal, reciprocal associations. Journal of clinical child and adolescent psychology: the official journal for the Society of Clinical Child and Adolescent Psychology, American Psychological Association, Division 53.

[CR50] Opsahl T, Agneessens F, Skvoretz J (2010). Node centrality in weighted networks: Generalizing degree and shortest paths. Social Networks.

[CR51] Pereira-Morales AJ, Adan A, Forero DA (2019). Network analysis of multiple risk factors for mental health in young Colombian adults. Journal of Mental Health.

[CR52] Pesola F, Shelton KH, Heron J, Munafò M, Hickman M, van den Bree MB (2015). The developmental relationship between depressive symptoms in adolescence and harmful drinking in emerging adulthood: The role of peers and parents. Journal of Youth and Adolescence.

[CR53] R Core Team. (2019). R: A language and environment for statistical computing. In *R Foundation for statistical computing*. Vienna: URL https://www.R-project.org/.

[CR54] Robinaugh DJ, Millner AJ, McNally RJ (2016). Identifying highly influential nodes in the complicated grief network. Journal of Abnormal Psychology.

[CR55] Schellekens, M. P. J., Wolvers, M. D. J., Schroevers, M. J., Bootsma, T. I., Cramer, A. O. J., & van der Lee, M. L. (2020). Exploring the interconnectedness of fatigue, depression, anxiety and potential risk and protective factors in cancer patients: a network approach. *J Behav Med, 43*(4):553–563. 10.1007/s10865-019-00084-7. 10.1007/s10865-019-00084-7 PMC7366596 31435892

[CR56] Schoevers RA, Deeg DJH, van Tilburg W, Beekman ATF (2005). Depression and generalized anxiety disorder: Co-occurrence and longitudinal patterns in elderly patients. The American Journal of Geriatric Psychiatry.

[CR57] Stapinski LA, Araya R, Heron J, Montgomery AA, Stallard P (2015). Peer victimization during adolescence: Concurrent and prospective impact on symptoms of depression and anxiety. Anxiety, Stress, & Coping.

[CR58] Starr LR, Davila J (2011). Responding to anxiety with rumination and hopelessness: Mechanism of anxiety-depression symptom co-occurrence?. Cognitive Therapy and Research.

[CR59] Stattin H, Kerr M (2000). Parental monitoring: A reinterpretation. Child Development.

[CR60] Steinley D, Hoffman M, Brusco MJ, Sher KJ (2017). A method for making inferences in network analysis: Comment on Forbes, Wright, Markon, and Krueger (2017). Journal of Abnormal Psychology.

[CR61] Terluin B, de Boer MR, de Vet HCW (2016). Differences in connection strength between mental symptoms might be explained by differences in variance: Reanalysis of network data did not confirm staging. PLoS One.

[CR62] Turner N, Joinson C, Peters TJ, Wiles N, Lewis G (2014). Validity of the short mood and feelings questionnaire in late adolescence. Psychological Assessment.

[CR63] Van Veen T, Wardenaar KJ, Carlier IVE, Spinhoven P, Penninx BWJH, Zitman FG (2013). Are childhood and adult life adversities differentially associated with specific symptom dimensions of depression and anxiety? Testing the tripartite model. Journal of Affective Disorders.

[CR64] Williams DR, Rast P (2019). Back to the basics: Rethinking partial correlation network methodology. British Journal of Mathematical and Statistical Psychology.

[CR65] Wolke D, Woods S, Stanford K, Schulz H (2001). Bullying and victimization of primary school children in England and Germany: Prevalence and school factors. British Journal of Psychology.

[CR66] Young CC, Dietrich MS (2015). SLEs, worry, and rumination predict depressive and anxiety symptoms in Young adolescents. Journal of Child and Adolescent Psychiatric Nursing.

