# Supplementary material for: Comorbidity Between Depression and Anxiety in Adolescents: Bridge Symptoms and Relevance of Risk and Protective Factors
Source: J Psychopathol Behav Assess. 2021 Mar 30;43(3):583–96. doi: 10.1007/s10862-021-09880-5 (PMC8550210; doi:10.1007/s10862-021-09880-5)
Supplement: Supplementary file 1 — (DOCX 2103 kb) [file 10862_2021_9880_MOESM1_ESM.docx]

**Supplementary Materials**

**Article Title:** Comorbidity Between Depression and Anxiety in Adolescents: Bridge Symptoms and Relevance of Risk and Protective Factors

**Journal Name:** Journal of Psychopathology and Behavioral Assessment

**Author Names:** Deniz Konac, Edward D Barker, Katherine S Young, Jennifer Lau

**Affiliation and Contact Information of the Corresponding Author:** King’s College London, [deniz.konac@kcl.ac.uk](mailto:deniz.konac@kcl.ac.uk)

**Included vs. Not-Included Sample:**

Unfortunately, there was a high number of missing data across the high number of measures (i.e. symptoms, risks and protective factors) used in the study. Also, the total ALSPAC sample size (n=15,440) when the cohort study first started had considerably decreased by the time participants reached 13 years of age (n=7,108). We included participants who had less than three missing items on both depression and anxiety measures when examining the symptoms. 112 participants did not provide enough data on depression measures, 192 on anxiety measures, 429 on overt victimization measures, 452 on relational victimization measures, 437 on overt bullying measures, 453 on relational bullying measures, 122 on peer relational problems measures, 122 on prosocial behavior measures, 1,518 on child disclosure measures, and 1,666 on parental knowledge measures. SLEs were measured at 11 years of age and 152 out of 7,596 participants who had provided any data at all at the given time point did not provide enough data for this study. After removing 112 participants on depression measure (6,996 remained), and 192 due to missingness on anxiety measure (6,916 remained), these remaining participants did not overlap perfectly. Hence, we further removed participants who provided enough data on the depression measure but not on the anxiety measure. This resulted in 6054 eligible participants (85.2% of total ALSPAC sample at age-13) who provided enough data on both measures. As can be seen, 14.8% of total ALSPAC sample at 166 months was hence removed due to missingness on depression and anxiety measures alone. In addition, the patterns of missing data between the symptoms and the risk and protective factors was also quite high. For example, 1,666 participants were removed due to missingness on parental knowledge measure (i.e. 1,666 participants had more than two missing items on parental knowledge measure, hence, 5,442 participants remained). Again, because the remaining sample after removing participants due to missingness on depression and anxiety measures (n=6,054) did not perfectly overlap with the remaining participants on parental knowledge measure (n=5,442), we further removed participants who provided enough data on depression and anxiety measures but not on parental knowledge measure, and vice versa. This resulted in a sample of 4,032 participants who provided enough data on these three measures. We further removed participants based on missingness on the rest of the measures used in the study (i.e. overt victimization, relational victimization, overt bullying, relational bullying, peer relational problems, stressful life events, prosocial behavior, and child disclosure measures) with the same method. As we included participants with complete data (i.e. no more than 2 missing items per measure), the included sample (n = 3,670) was considerably small compared to the total ALSPAC sample at age 13 (n=7,108). The visualization of missing data pattern is presented in S1.

**General Structure of The Networks**

In both steps, depression symptoms and anxiety symptoms had several bridge edges, however, symptoms that belong to the same disorder had more and stronger edges between each other. While it was apparent from this network structure that depression and anxiety symptoms were interconnected, they still do not give the impression of forming one single disorder. Previous studies yielded to mixed results on this matter, as another study also found within disorder associations were stronger than between disorder associations (Beard et al., 2016) while other studies found within disorder and between disorder associations were of similar strength (McElroy et al., 2018; Cramer et al., 2010; Curtiss & Klemanski, 2016). Of note, the time difference of 6 months between the administration of depression and anxiety measurements might have led to sparser associations between the symptoms of these disorders.

**Centrality Indices**

Centrality indices of strength, closeness, and expected influence were found to be stable in step-1; and strength, closeness, betweenness, and expected influence were found to be stable in step-2, based on the CS-coefficients. Relevant CS-coefficients indicating the stability of these centrality indices are presented in S2 and significant differences between symptom rankings on these indices are presented in S3-S5.

**Centrality Properties of the Symptoms**

Ranking of depression and anxiety symptoms on these centrality indices were comparable and the most central symptoms in the networks belonged to both disorders. Similarly, the strength of within disorder associations between the symptoms of both disorders were also comparable. These results suggest that symptoms of neither disorder exerted more influence on the overall networks and both disorders were equally important.

Symptoms rank higher on centrality indices in a network are believed to have a strong impact on less central nodes, deeming them both risk factors for developing further symptoms and the best targets for intervention (Fried et al., 2017; Fried et al., 2016). Indeed, previous research showed central symptoms to be the most predictive symptoms of future disorder onset (Boschloo et al., 2016). Among the depression symptoms examined in this study, the symptoms relating to feelings of worthlessness (“feeling self is no good anymore”, “hating self”, “feeling like they did everything wrong”) and the symptom of “feeling unhappy” were found to have the highest centralities in both steps, when all stable centrality indices are taken into consideration. “Feeling unhappy” also emerged as a prominent bridge symptom. These results imply that anhedonia and strong feelings of worthlessness may be the main factors triggering the onset and/or persistence of depressive disorders and targeting these symptoms may result in an overall reduction in depression severity. This is in line with previous studies that also found symptoms related to feelings of worthlessness and anhedonia to be central depression symptoms (Hereen et al., 2018; Mullarkey et al., 2018; Beard et al., 2016; Langer et al., 2019).

Among the anxiety symptoms examined in this study, “worrying about bad things happening to others” and “worrying about school” were the most central in both networks, when all stable centrality indices are considered. That is somewhat contrary to McElroy and colleagues’ (2018) findings, as the authors found “fears school” to be one of the least central nodes. This may be due to the slight difference in what “fearing school” and “worrying about school” refer to. Alternatively, as stated by the authors, order of the centrality indices in the study may not be reliable given the low stability of the centrality indices, hence these rankings may not necessarily reflect the true relative importance of the symptoms.

In sum, depression symptoms that reflect feelings of worthlessness (“feeling self is no good anymore”, “hating self”, “feeling like they did everything wrong”) and anhedonia (“feeling unhappy”); and anxiety symptoms that are related to academic difficulties and safety of others were the most central symptoms in both networks. Accordingly, targeting these symptoms may lead to an overall reduction in severity of depression and anxiety.

**Centrality Properties of the Risk/Protective Factors**

All risk/protective factors were less central than the above-mentioned highly central symptoms. This suggest that while some risk/protective factors ranked high on centrality indices, they were still less influential than the most central symptoms in the network.

The most central risk factor was overt victimization, which was strongly associated with risk factors of relational victimization and overt bullying. Overt victimization was also associated with risk factors of SLEs and peer relational problems, and depression symptom of “feeling unhappy.” Peer relational problems was the risk factor that ranked highest on betweenness and closeness centrality indices. Importantly, betweenness centrality indicates the number of times a node lies on the shortest path between any other two nodes and closeness centrality indicates the average edge distance from one node to all other nodes (Jones et al., 2019). Thus, this result suggests that peer relational problems connects several symptoms and other risk/protective factors with each other and contribute to comorbidity. Indeed, it is apparent from the network in step-2 that peer relational problems has several associations with depression and anxiety symptoms, as well as with SLEs and overt victimization.

The most central protective factors were parental knowledge and child disclosure, which had associations with several depression/anxiety symptoms and risk/protective factors. These protective factors were negatively associated with overt bullying, indicating an important pathway as to how these factors exert a protective effect against risk factors. Interestingly, child disclosure was positively associated with depression symptom of “crying a lot” and anxiety symptom of “worrying about school.” This suggests adolescents who experience these symptoms may be disclosing more information to their parents. In addition, protective factor of prosocial behaviour was the highest-ranking protective factor on betweenness centrality, indicating this factor played an important role in associating other nodes with each other.

Overall, these results depict the importance of peer victimization in influencing depression/anxiety symptom severity and the other risk/protective factors. Similarly, parental knowledge and child disclosure appears to have moderate influence on the other variables in the network. Finally, while risk factor of peer relational problems and protective factor of prosocial problem did not exert strong influence on other nodes in the network per se, they were highly functional in associating different nodes with each other.

**Supplementary Figures**

**
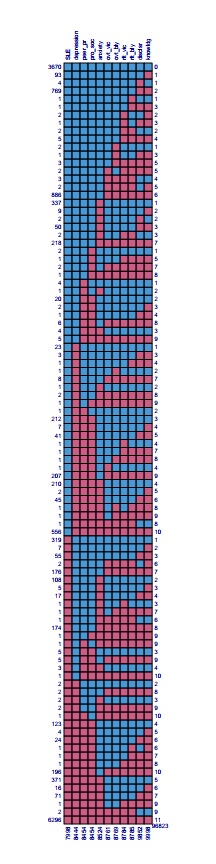
**

**Figure S1. Missingness Pattern.** Missingness pattern at age-13 shown against the original ALSPAC sample (n=15,440). Blue boxes represent participants who provided enough data (a maximum of two missing items per measure), and red boxes represent participants who did not provide enough data (more than two missing items per measure). Only the participants who provided enough data (a maximum of two missing items per measure) were included to the analysis sample (i.e. the first row of the plot, n=3,670). The numbers on the left-hand side show the number of participants with the given pattern. The numbers on the right-hand side show the number of measures with insufficient data. The numbers at the bottom show the total number of participants excluded due to missingness on the given measure. Measures are represented as the column names in the plot.

**Figure S2. Bootstrapped 95% Confidence Intervals of the Edge Weights for Network in Step-1**. Red dots represent the value of edge weight, and grey lines represent bootstrapped 95% confidence interval.


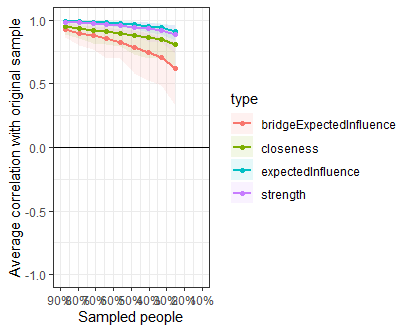


**Figure S3. Case-Dropping Bootstrapped Centrality Indices for Network in Step-1.** Lines reflect mean correlations between centrality values of original sample and sub samples with different degrees of persons dropped, and areas around the lines reflect 95% CIs.


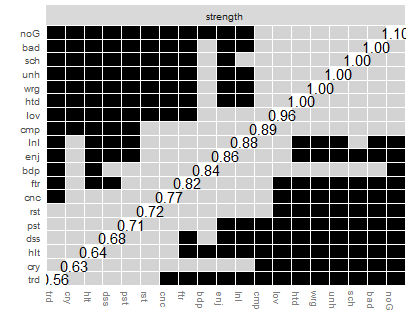


**Figure S4. Differences in Strength Centrality in Step-1.** Black squares depict a significant difference in strength centrality between two nodes and grey squares depict a non-significant difference between two nodes.


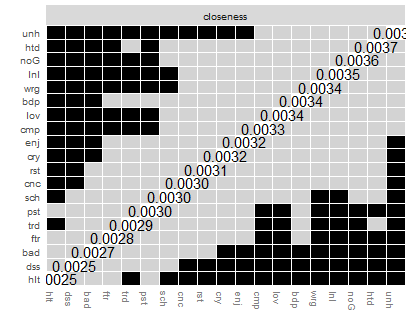


**Figure S5. Differences in Closeness Centrality in Step-1.** Black squares depict a significant difference in strength centrality between two nodes and grey squares depict a non-significant difference between two nodes.


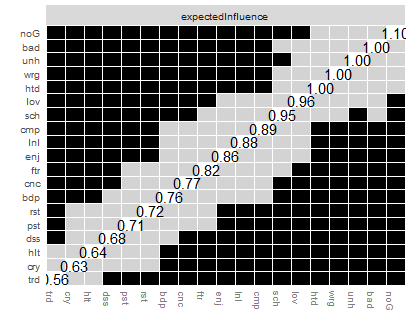


**Figure S6. Differences in Expected Influence Centrality in Step-1.** Black squares depict a significant difference in strength centrality between two nodes and grey squares depict a non-significant difference between two nodes.


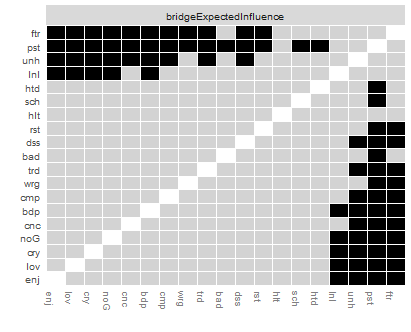


**Figure S7. Differences in Bridge Expected Influence Centrality in Step-1.** Black squares depict a significant difference in strength centrality between two nodes and grey squares depict a non-significant difference between two nodes.


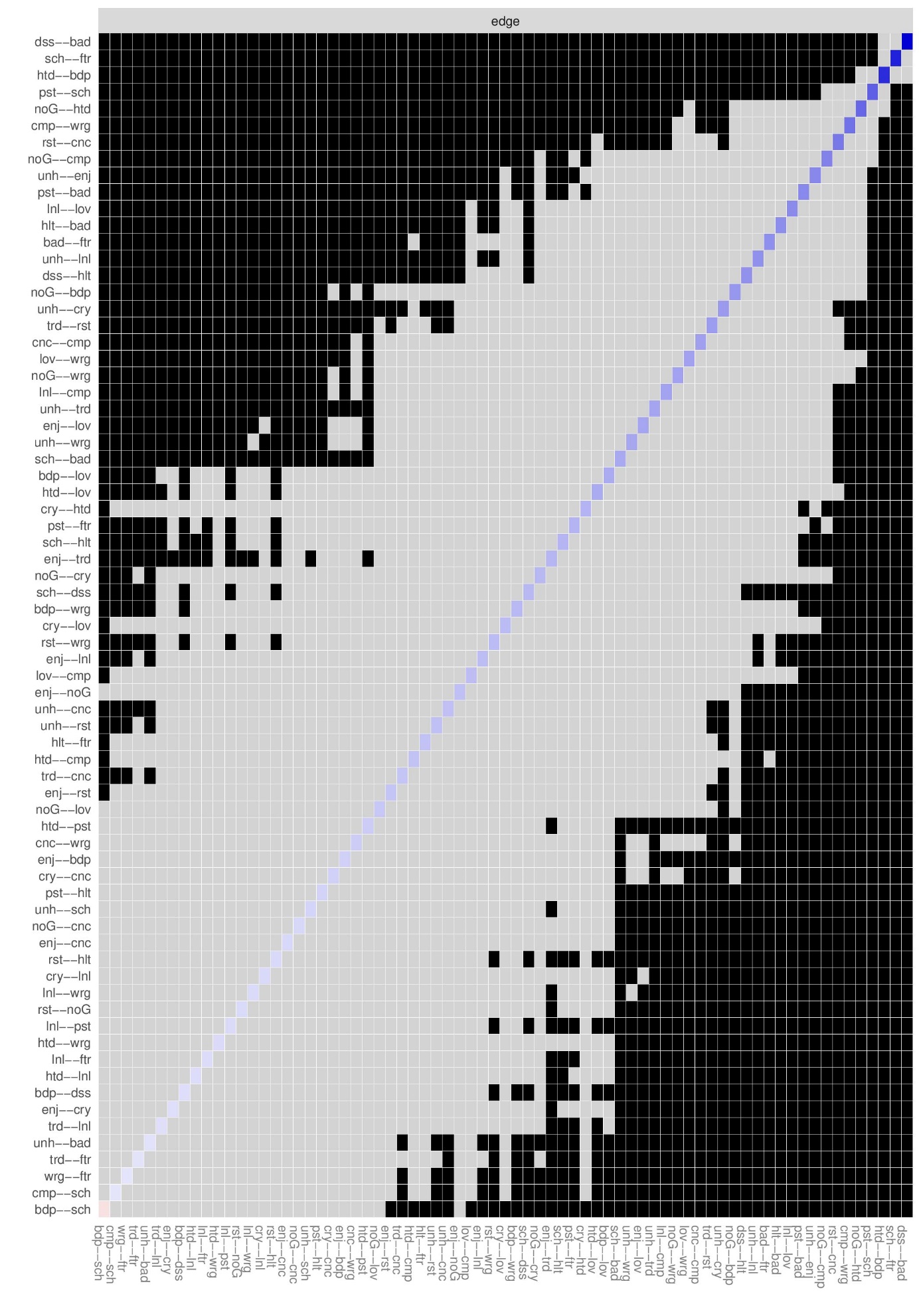


**Figure S8. Bootstrapped Edge Difference Test for Network in Step-1**. Black squares depict a significant difference between the edge weights and grey squares depict a non-significant difference.

**Figure S9. Bootstrapped 95% Confidence Intervals of the Edge Weights for Network in Step-2**. Red dots represent the value of edge weight, and grey lines represent bootstrapped 95% confidence interval.


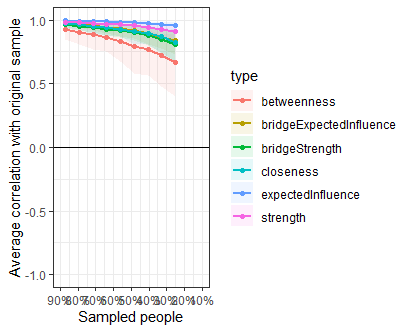


**Figure S10. Case-Dropping Bootstrapped Centrality Indices for Network in Step-2.** Lines reflect mean correlations between centrality values of original sample and sub samples with different degrees of persons dropped, and areas around the lines reflect 95% CIs.


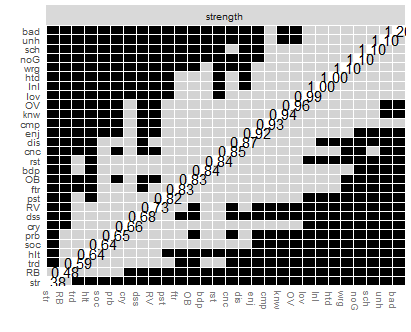


**Figure S11. Differences in Strength Centrality in Step-2.** Black squares depict a significant difference in strength centrality between two nodes and grey squares depict a non-significant difference between two nodes.


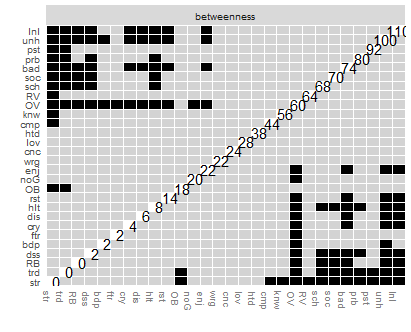


**Figure S12. Differences in Betweenness Centrality in Step-2.** Black squares depict a significant difference in strength centrality between two nodes and grey squares depict a non-significant difference between two nodes.


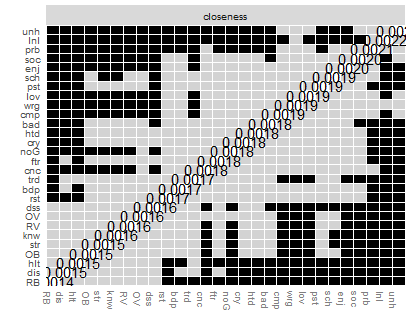


**Figure S13. Differences in Closeness Centrality in Step-2.** Black squares depict a significant difference in strength centrality between two nodes and grey squares depict a non-significant difference between two nodes.


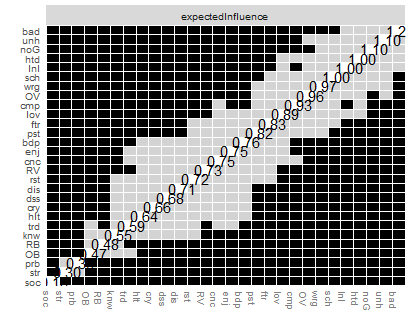


**Figure S14. Differences in Expected Influence Centrality in Step-2.** Black squares depict a significant difference in strength centrality between two nodes and grey squares depict a non-significant difference between two nodes.


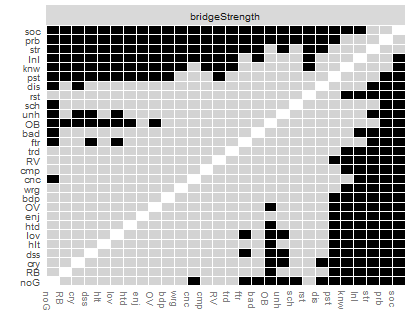


**Figure S15. Differences in Bridge Strength Centrality in Step-2.** Black squares depict a significant difference in strength centrality between two nodes and grey squares depict a non-significant difference between two nodes.


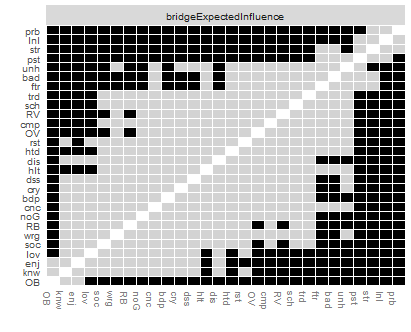


**Figure S16. Differences in Bridge Expected Influence Centrality in Step-2.** Black squares depict a significant difference in strength centrality between two nodes and grey squares depict a non-significant difference between two nodes.


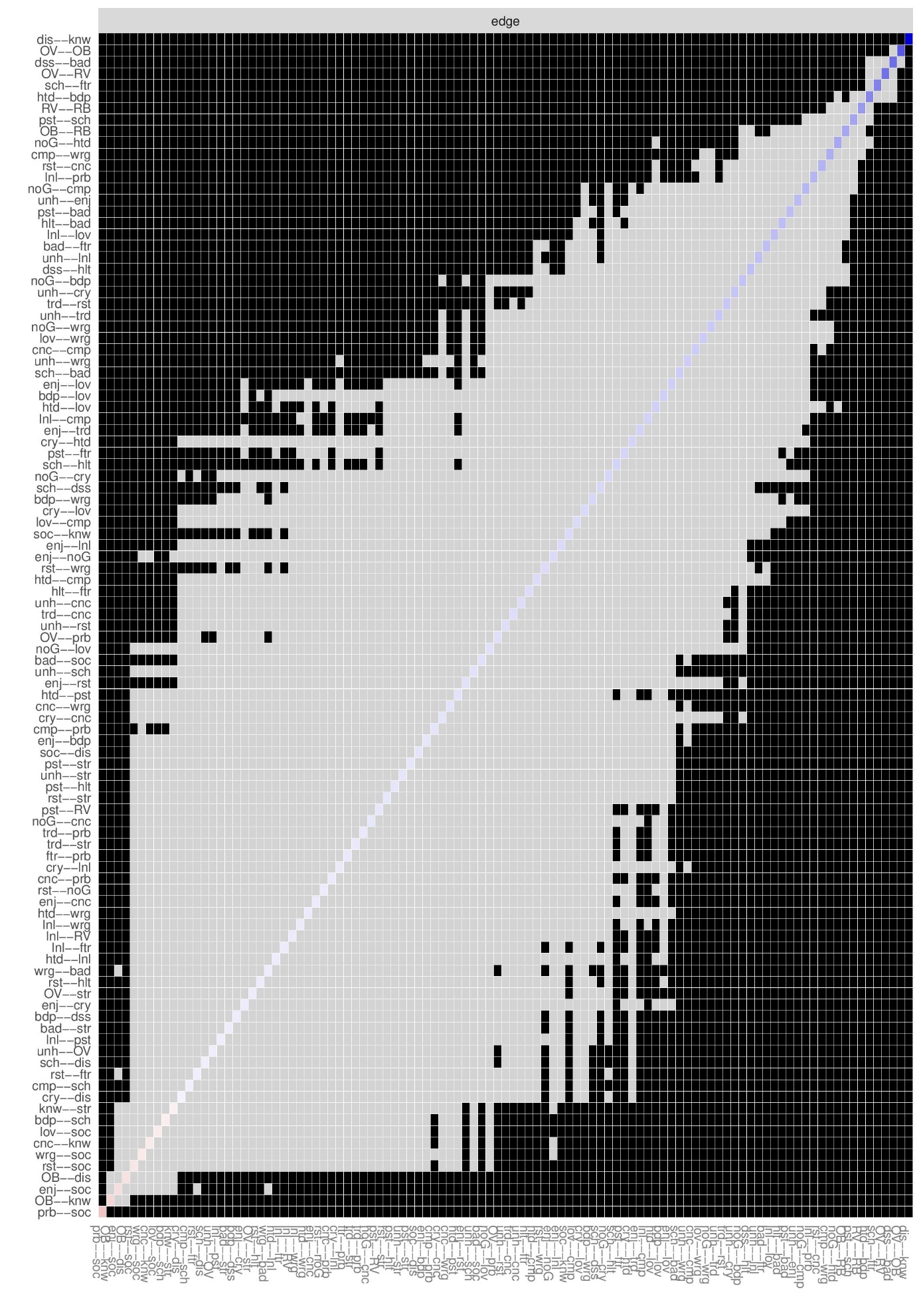


**Figure S17. Bootstrapped Edge Difference Test for Network in Step-2**. Black squares depict a significant difference between the edge weights and grey squares depict a non-significant difference.

**References**

Beard, C., Millner, A. J., Forgeard, M. J. C., Fried, E. I., Hsu, K. J., Treadway, M. T., … Björgvinsson, T. (2016). Network analysis of depression and anxiety symptom relationships in a psychiatric sample. Psychological Medicine, 46(16), 3359–3369.

Boschloo, L., Schoevers, R. A., van Borkulo, C. D., Borsboom, D., & Oldehinkel, A. J. (2016). The network structure of psychopathology in a community sample of preadolescents. Journal of Abnormal Psychology, 125(4), 599–606.

Cramer AOJ, Waldorp LJ, van der Maas HLJ, Borsboom D (2010) Comorbidity: a network perspective. Behav Brain Sci 33(2–3):137–150.

Curtiss, J., & Klemanski, D. H. (2016). Taxonicity and network structure of generalized anxiety disorder and major depressive disorder: An admixture analysis and complex network analysis. Journal of Affective Disorders, 199, 99–105.

Fried EI, Epskamp S, Nesse RM, Tuerlinckx F, Borsboom D (2016) What are ‘‘good’’ depression symptoms? Comparing the centrality of DSM and non-DSM symptoms of depression in a network analysis. J Affect Disord 189:314–320.

Fried, E.I., van Borkulo, C.D., Cramer, A.O.J. et al. Mental disorders as networks of problems: a review of recent insights. Soc Psychiatry Psychiatr Epidemiol 52, 1–10 (2017).

Heeren, A., Jones, P. J., McNally, R. J. (2018). Mapping network connectivity among symptoms of social anxiety and comorbid depression in people with social anxiety disorder. Journal of Affective Disorders. 228, 75-82.

Jones, P.J, Ma, R. & McNally, R.J. (2019): Bridge Centrality: A Network Approach to Understanding Comorbidity, Multivariate Behavioral Research.

Langer, J. K., Tonge, N. A., Piccirillo, M., Rodebaugh, T. L., & Thompson, R. J. (2018). Symptoms of Social Anxiety Disorder and Major Depressive Disorder: A Network Perspective. Journal of Affective Disorders, 243, 531-538.

McElroy, E., Fearon, P., Belsky, J., Fonagy, P. & Patalay, P. Networks of Depression and Anxiety Symptoms Across Development. Journal of the American Academy of Child & Adolescent Psychiatry, Volume 57, Issue 12, 964 – 973.

Michael C. Mullarkey, Igor Marchetti & Christopher G. Beevers (2018): Using Network Analysis to Identify Central Symptoms of Adolescent Depression, Journal of Clinical Child & Adolescent Psychology, 00(00), 1–13.
